# Does endometrioma diameter impact serum anti-Müllerian hormone levels? A systematic review and meta-analysis

**DOI:** 10.1093/hropen/hoag048

**Published:** 2026-05-22

**Authors:** Johnny S Younis, Liana Glick, Pietro Santulli, Ido Izhaki, Nora Shapso

**Affiliations:** Reproductive Medicine, Department of Obstetrics and Gynecology, Tzafon Medical Center, Poriya, Israel; Faculty of Medicine, Bar-Ilan University, Safed, Israel; Faculty of Medicine, Bar-Ilan University, Safed, Israel; Faculté de Santé, Université Paris Cité, Paris, France; Department of Gynecology and Obstetrics II and Reproductive Medicine (Professor Chapron), Assisstance Publique-Hôpitaux de Paris (AP-HP), Hôpital Universitaire Paris Centre (HUPC), Centre Hospitalier Universitaire (CHC), Paris, France; Department of Evolutionary and Environmental Biology, University of Haifa, Haifa, Israel; Reproductive Medicine, Department of Obstetrics and Gynecology, Tzafon Medical Center, Poriya, Israel

**Keywords:** anti-Müllerian hormone, endometrioma, endometriosis, endometrioma diameter, fertility preservation, mass effect, meta-regression, ovarian reserve, surgical threshold

## Abstract

**STUDY QUESTION:**

Is endometrioma diameter associated with ovarian reserve, as reflected by serum anti-Müllerian hormone (AMH) levels, in women of reproductive age with no history of ovarian surgery?

**SUMMARY ANSWER:**

Across 30 independent cohorts, endometrioma diameter showed no independent association with serum AMH levels when modeled either continuously or categorically, indicating that cyst size is unlikely to be a principal determinant of diminished ovarian reserve in non-operated endometrioma.

**WHAT IS KNOWN ALREADY:**

The extent to which endometrioma diameter contributes to ovarian reserve impairment remains uncertain and has historically informed size-based thresholds for surgical intervention. Whether larger cysts exert a greater deleterious effect on ovarian reserve remains debated. Observational studies in women without prior ovarian surgery have reported inconsistent and often contradictory findings.

**STUDY DESIGN, SIZE, DURATION:**

Systematic review and meta-analysis of studies published in English between 1 January 2000 and 30 June 2025. Searches were conducted in PubMed, ClinicalTrials.gov, the Cochrane Library, Web of Science, and EBSCOhost.

**PARTICIPANTS/MATERIALS, SETTING, METHODS:**

Eligible studies compared serum AMH across different endometrioma diameters within the same population and setting in reproductive-age women with non-operated endometrioma. Two reviewers independently screened studies, extracted data, and assessed risk of bias using ROBINS-I. The primary inferential analysis was a cohort-level multivariable random-effects meta-regression based on 30 independent, non-overlapping cohorts derived from 16 studies, adjusting for age. Diameter was modeled both as a continuous predictor and as prespecified strata (<4 cm, 4–5 cm, 5–6 cm, >6 cm). Supportive exploratory threshold and subgroup analyses (predefined cut-offs 3–8 cm) were interpreted with multiplicity control using the Holm–Bonferroni sequentially rejective procedure.

**MAIN RESULTS AND THE ROLE OF CHANCE:**

Sixteen studies, including 1484 women, met the inclusion criteria, and 30 independent cohorts were used in the primary analysis. Endometrioma diameter was not a significant predictor of serum AMH when modeled continuously (*β* = 0.122, SE = 0.128, *P *= 0.35) or categorically across diameter strata (*β* = 0.245, SE = 0.220, *P *= 0.28). In contrast, age accounted for most of the between-cohort heterogeneity (*R*^2^ = 58.2%). Supportive subgroup and threshold analyses did not materially alter the primary inference after multiplicity adjustment. Of the 16 eligible studies, 2 were judged to be at low risk and 14 at moderate risk of bias, suggesting overall moderate certainty of the evidence.

**LIMITATIONS, REASONS FOR CAUTION:**

The analysis was restricted to non-operated endometriomas; therefore, the relationship between preoperative diameter and post-surgical AMH decline could not be assessed. Residual confounding is possible given the observational nature of the included studies and variation in measurement and laboratory methods.

**WIDER IMPLICATIONS OF THE FINDINGS:**

In women with non-operated endometrioma, ovarian reserve impairment, when present, appears largely independent of cyst diameter, arguing against a predominant ‘mass effect’ as the principal mechanism. These findings support clinical counseling and management strategies that do not rely on cyst size alone when considering infertility care, delayed childbearing, and fertility preservation.

**STUDY FUNDING/COMPETING INTEREST(S):**

No funding was received for the conduct of this study. The authors declare no competing interests.

**REGISTRATION NUMBER:**

Prospectively registered at PROSPERO under number CRD42025632149 on 28 December 2024.

WHAT DOES THIS MEAN FOR PATIENTS? If a patient has been diagnosed with an ovarian cyst from endometriosis (an endometrioma), they might worry that a larger cyst is doing more damage to their ‘egg count’ (ovarian reserve) or future fertility. For a long time, doctors believed in a ‘mass effect’, the idea that a large cyst physically crushes or stretches the ovary, causing more harm than a small one. Because of this, many women were told they needed surgery simply because their cyst reached a certain size, like 4 cm.

Our study looked at data from nearly 1500 women and found that the size of the cyst does not actually predict their egg count. Whether a cyst is small or large, the impact on their ovarian reserve appears to be the same. This means that a larger cyst is not necessarily more ‘dangerous’ to their fertility than a small one, and the physical size alone should not be the only reason to have surgery. Instead, decisions about their care should be based on their age, their symptoms (like pain), and their personal goals for having children. These findings can support clearer counseling and more personalized decisions about fertility planning, delayed childbearing, and fertility preservation.

## Introduction

Endometriosis is a globally prevalent gynecological disorder, affecting nearly 1 in 10 women of reproductive age, with an estimated 200 million cases worldwide (Zondervan *et al.*, 2020). The disease features the presence of ectopic endometrial-like tissue outside the uterine cavity, which is estrogen-dependent and often exhibits resistance to progesterone. This aberrant tissue triggers a chronic inflammatory response, which can have detrimental effects on adjacent organs (Marquardt *et al.*, 2019; Taylor *et al.*, 2021). A distinct diagnosed type of the disease is the ovarian endometrioma, which may be indicative of advanced-stage endometriosis (American Society For Reproductive Medicine, 1997; Guerriero *et al.*, 2016). Epidemiological data suggest that unilateral and bilateral endometriomas are observed in up to 55% and 28% of affected individuals, respectively (Jenkins *et al.*, 1986; Vercellini *et al.*, 1998).

The mechanisms by which endometrioma impairs fertility and depletes ovarian reserve remain incompletely elucidated. Current evidence points to chronic inflammation, oxidative stress, cortical fibrosis, and follicular attrition or burnout as principal culprits (Kitajima *et al.*, 2011; Sanchez *et al.*, 2014; Wu *et al.*, 2019; Wyatt *et al.*, 2023). In parallel, a mechanical component such as pressure, stretching, or distortion of the ovarian cortex by the endometrioma itself has long been suspected (Maneschi *et al.*, 1993; Kitajima *et al.*, 2011; Leone Roberti Maggiore *et al.*, 2017). However, whether these mechanisms increase in proportion to endometrioma size remains unresolved. This is not only of pathophysiological interest but also of direct clinical relevance in reproductive-aged women, especially those facing infertility or delayed childbearing. In this context, choosing between expectant, medical, or surgical management of endometrioma requires weighing potential benefits against the risks to ovarian reserve.

Furthermore, although the impact of an intact endometrioma on ovarian reserve remains debated (Streuli *et al.*, 2012; Benaglia *et al.*, 2017; Kasapoglu *et al.*, 2018; Muzii *et al.*, 2018), growing evidence indicates that endometriotic cystectomy may significantly impair ovarian reserve, with greater loss seen in cases of bilateral disease or repeat surgery (Raffi *et al.*, 2012; Ferrero *et al.*, 2015; Muzii *et al.*, 2015; Younis *et al.*, 2019). Among available markers, recent data suggest that in the context of endometrioma surgery, serum anti-Müllerian hormone (AMH) is a more sensitive measure than antral follicle count (AFC) for assessing ovarian reserve in women with endometrioma (Younis *et al.*, 2022). However, whether the size of endometrioma itself influences AMH levels remains a matter of active debate.

Over the past decade, studies examining this relationship have produced conflicting results. While some studies have identified a negative correlation between endometrioma size and serum AMH levels (Ergun *et al.*, 2015; Karadağ *et al.*, 2020), others have found no change (Hirokawa *et al.*, 2011; Bourdon *et al.*, 2022). Adding further complexity, additional independent studies observed increased serum AMH levels in women with larger endometriomas (Marcellin *et al.*, 2019; Roman *et al.*, 2021).

Taken together, these findings underscore persistent uncertainty regarding the impact of endometrioma size on ovarian reserve. Many previous studies suffer from methodological limitations, including small sample sizes, heterogeneous inclusion criteria, and potential sampling bias. A targeted systematic review and meta-analysis are therefore warranted to clarify this relationship. High-quality evidence would clarify the pathophysiological impact of endometrioma size on ovarian reserve, providing valuable clinical insights. Such evidence would support informed decision-making for women, particularly those experiencing infertility or planning future pregnancies. It could also guide patient counseling regarding treatment timing and oocyte preservation strategies.

This systematic review and meta-analysis aims to evaluate the impact of endometrioma diameter on ovarian reserve, as measured by serum AMH levels, in women without prior ovarian surgery.

## Methods

We conducted our systematic review and meta-analysis in accordance with the MOOSE statement, the updated PRISMA 2020 guidelines, and adhered to the latest version of the Cochrane Handbook for Systematic Reviews of Interventions (Stroup *et al.*, 2000; Page *et al.*, 2021; Higgins *et al.*, 2024). The study protocol was registered in advance with PROSPERO (International Prospective Register of Systematic Reviews) under registration number CRD42025632149.

### Study design

We conducted a comprehensive electronic database search using PubMed, ClinicalTrials.gov, the Cochrane Library, as well as Web of Science and EBSCO (as interfaces to databases) to identify research articles published between 1 January 2000 and 30 June 2025. EBSCOhost (short for Elton B. Stephens Company) is a large information platform that provides the ‘digital backbone’ for libraries worldwide. Our focus was on studies that examined the impact of ovarian endometrioma diameter on serum AMH levels in women of reproductive age. The systematic review and meta-analysis aimed to identify studies assessing serum AMH levels in women of reproductive age with varying endometrioma diameters, who had not undergone ovarian surgery, within the same population and setting.

The literature search was confined to studies involving human subjects, and we included only full-text articles published in peer-reviewed English-language journals. The inclusion criteria consisted of studies that evaluated serum AMH levels based on endometrioma diameter in women of reproductive age who had not undergone surgery or other minimally invasive procedures. Eligible study designs included randomized controlled trials, case–control studies, cohort studies, and observational studies.

To enhance data quality and reduce heterogeneity, we applied several exclusion criteria: studies involving pregnant women, women post-ovarian surgery (such as cystectomy or oophorectomy), ovarian cysts other than endometrioma, those who had undergone ablative or sclerotherapy procedures, or those who had received chemotherapy or radiotherapy. Additionally, we omitted studies that included women with polycystic ovary syndrome and other endocrine disorders, such as hyperprolactinemia and thyroid dysfunction, or women with irregular menstrual cycles, as well as those with a history of ovarian failure or pre-menopausal FSH levels. Studies that involved women taking oral contraceptives or other hormonal therapies at least 3 months before recruitment were also excluded. Furthermore, data presented solely as abstracts or at scientific meetings, as well as case reports, case series, reviews, perspectives, and opinion papers, were not considered.

### Search strategy

We used the following search terms to find eligible studies on search engines or database interfaces: (Endometrioma OR endometriosis OR endometriotic) AND (cyst OR ovarian OR size OR diameter OR volume OR effect OR diagnosis) AND (AMH OR anti-Müllerian OR antiMüllerian OR Müllerian inhibiting factor OR Müllerian inhibiting substance OR reserve).

### Study selection

We carried out the study selection in two stages. In the first stage, we screened the titles and abstracts of the queried articles to determine their suitability. Two review authors, L.G. and N.S., independently selected appropriate abstracts for full-text evaluation. In the second stage, we chose eligible studies for inclusion in the quantitative analysis. Any disagreements regarding study inclusion in both stages were resolved through discussion and consensus or, if necessary, mediation by a third reviewing author (J.S.Y.). We also manually reviewed the references of eligible full-text studies to identify any additional relevant publications. To prevent multiple inclusion of the same data, we selected the latest or largest study and excluded overlapping studies.

When data were missing, we emailed the first and last authors of the relevant publications, or the corresponding author, at least twice on separate occasions, with a minimum interval of 2 weeks between attempts, to request additional data.

### Data extraction

We extracted data from eligible studies using a standardized Excel template prepared by the review group. Our focus included the study’s methodology, population, and design, number of participating women, women’s ages, endometrioma diameter and laterality, serum AMH levels, AMH assay methodology, and data for evaluating risk of bias in each study.

Serum AMH levels were standardized to ng/ml, using a conversion factor of 7.14 for values reported in pmol/l. For studies that reported only medians, ranges, and sample sizes, we applied established formulas for meta-analyses to estimate means and SDs (Hozo *et al.*, 2005). When numerical AMH values were not explicitly provided, data were extracted from published figures.

Two authors (L.G. and N.S.) independently collected data from the eligible articles. When disagreements arose, the final decision was reached through discussion and consensus, or through mediation by a third reviewer (J.S.Y.).

### Assessment of risk of bias

To assess the risk of bias in the included non-randomized studies, we employed the Risk Of Bias In Non-randomized Studies of Interventions (ROBINS-I) tool, developed by the Cochrane Bias Methods Group (Sterne *et al.*, 2016). This tool is recommended as a rigorous method for assessing the risk of bias in conducting systematic reviews (Schünemann *et al.*, 2019). ROBINS-I is a comprehensive framework designed to evaluate potential bias in observational studies across seven domains: confounding, selection of participants, classification of interventions, deviations from intended interventions, missing data, measurement of outcomes, and selection of the reported result. Each study was independently assessed by two reviewers (N.S. and J.S.Y.) and categorized as having low, moderate, serious, or critical risk of bias in each domain. Discrepancies were resolved through discussion or consultation with a third reviewer (I.I.). This approach enabled a structured and transparent evaluation of internal validity, accounting for the methodological heterogeneity inherent to observational study designs.

### Data analysis and statistical methods

Continuous variables (age and AMH) were compared between laterality groups (unilateral vs unilateral/bilateral combined) using independent-samples *t*-tests, based on aggregated study-level data.

### Primary multivariable meta-regression

The primary inferential analysis consisted of a cohort-level multivariable meta-regression including 30 independent, non-overlapping cohorts derived from the 16 eligible studies. Cohort independence was ensured by extracting mutually exclusive subgroups as defined in the original publications.

Associations between endometrioma diameter and serum AMH concentrations were evaluated using two complementary modeling strategies:

1. Continuous specification: Mean cyst diameter (cm) was entered as a continuous predictor to assess linear associations.
2. Categorical specification**:** Diameter was categorized into four prespecified ordinal strata (<4 cm, 4–5 cm, 5–6 cm, >6 cm) to evaluate potential non-linear or threshold effects. Categories were defined to ensure balanced representation across cohorts.

Both models were adjusted *a priori* for mean maternal age, given its established biological relationship with ovarian reserve and its potential contribution to between-cohort heterogeneity.

Random-effects meta-regression models were estimated using Restricted Maximum Likelihood (REML), providing conservative effect estimates while accounting for anticipated clinical and methodological variability across studies.

### Secondary and exploratory analyses

Supportive secondary analyses were conducted using predefined diameter cut-offs (3–8 cm) based on groupings reported in the 16 eligible studies. As these comparisons involve repeated use of overlapping cohorts, they were prespecified as exploratory and hypothesis-generating.

To assess the robustness of findings and explore potential sources of heterogeneity, subgroup analyses were performed according to key study characteristics, including study design, disease laterality, study population, AMH assay specification, sample processing strategy, and cyst measurement methodology.

Multiplicity arising from repeated comparisons was controlled using the Bonforroni–Holm sequentially rejective procedure, maintaining a stringent family-wise error rate without excessive loss of statistical power. Statistical significance was defined as two-tailed *P *< 0.05 after adjustment for multiplicity where applicable.

### Heterogeneity and statistical assessment

Between-study heterogeneity was evaluated using Cochran’s Q statistic and quantified with the *I*^2^ metric (Higgins *et al.*, 2003). Given the limited number of studies in certain analyses, interpretation of Q statistics was supplemented by *I*^2^ estimates, recognizing that Q may lack power in small meta-analyses.

All analyses were conducted using SPSS Statistics version 29 (IBM Corp., Armonk, NY, USA) and OpenMeta [Analyst], available at www.cebm.brown.edu/openmeta.

## Results

### Search results and excluded studies

**Figure 1.**
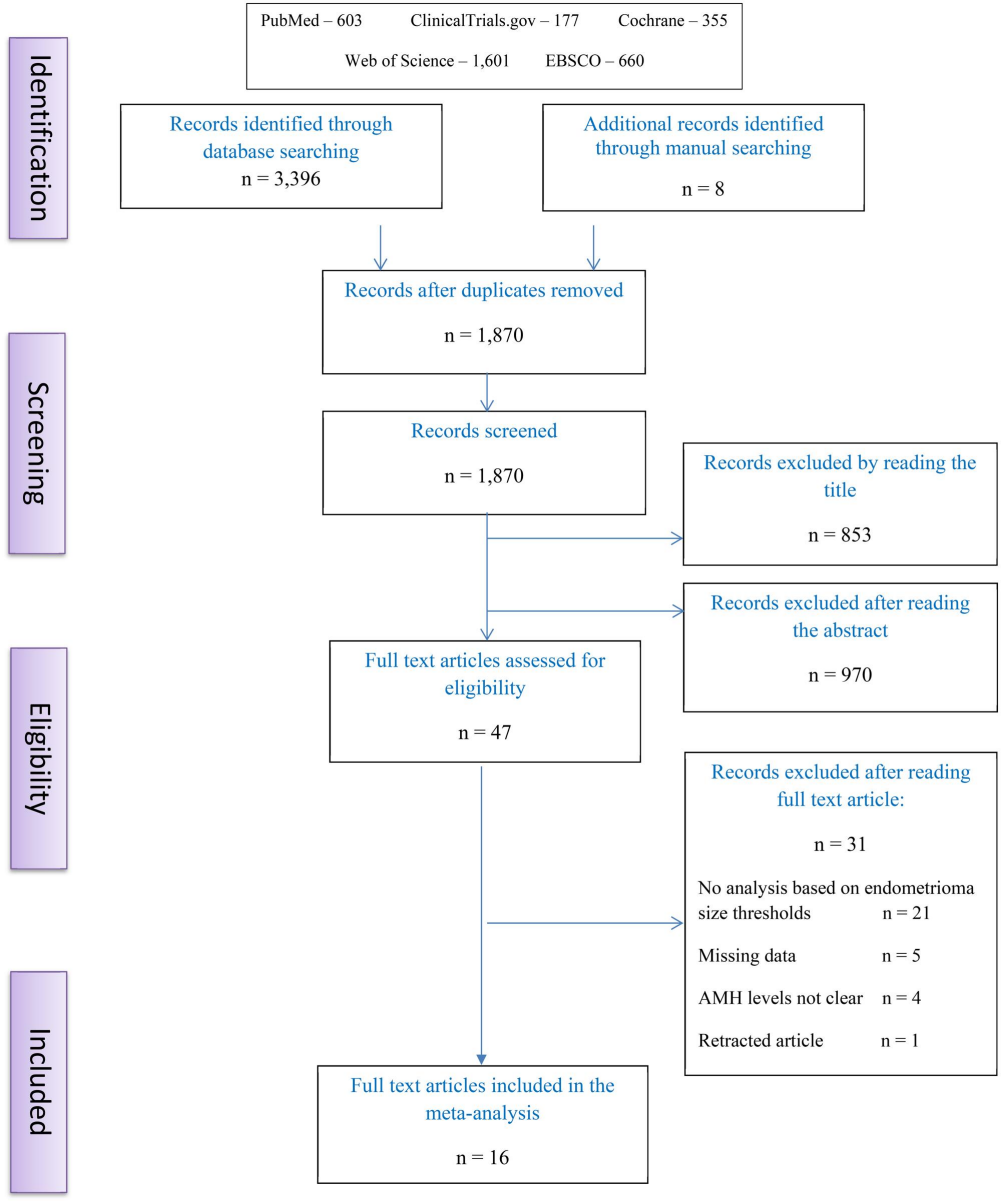
**Flow diagram of identified studies**.

Our search identified 3404 studies, of which 1870 were duplicates; 853 were excluded after reviewing the titles, and 970 after reading the abstracts (Fig. 1). Of the final 47 full-text articles assessed for eligibility, 31 were excluded from further evaluation because they did not meet the inclusion and exclusion criteria. In 21 studies, the impact of endometrioma size on serum AMH levels was not assessed (Ercan *et al.*, 2010, 2011; Tsolakidis *et al.*, 2010; Lee *et al.*, 2011; Litta *et al.*, 2013; Uncu *et al.*, 2013; Urman *et al.*, 2013; Aboul Gheit *et al.*, 2014; Chen *et al.*, 2014; Kwon *et al.*, 2014; Saito *et al.*, 2014, 2018; Tanprasertkul *et al.*, 2014; Ottolina *et al.*, 2017; Candiani *et al.*, 2018; Kasapoglu *et al.*, 2018; Kostrzewa *et al.*, 2019; Muzii *et al.*, 2019; Sweed *et al.*, 2019; Weng *et al.*, 2023; Azizova *et al.*, 2024).

Five studies examined endometrioma diameter cutoffs in relation to serum AMH levels, but essential data were missing, preventing their inclusion in the quantitative review (Abd Elhameid *et al.*, 2018; Dong *et al.*, 2019; Yilmaz *et al.*, 2021; Grigalashvili, 2022; Mansouri *et al.*, 2022). In four studies, serum AMH levels were not clearly reported (Ding *et al.*, 2015; Marcellin *et al.*, 2019; Elizur *et al.*, 2023; Kang *et al.*, 2023). One publication was retracted (Alborzi *et al.*, 2014).

The authors of 17 publications were contacted by email at least twice to request missing data; only one responded and provided the data, which were included in the quantitative analysis (Bourdon *et al.*, 2022).

### Included studies

A total of 16 studies (Table 1), involving 1484 women of reproductive age were included in the quantitative analysis, with a weighted mean age and weighted SD of 31.54 ± 2.18 years (Hirokawa *et al.*, 2011; Celik *et al.*, 2012; Ergun *et al.*, 2015; Giampaolino *et al.*, 2015; Javedani Masroor *et al.*, 2015; Sandya and Kumar, 2016; Mehdizadeh Kashi *et al.*, 2017; Wang *et al.*, 2019; Karadağ *et al.*, 2020; Yoon *et al.*, 2020; Roman *et al.*, 2021; Akgul *et al.*, 2022; Bourdon *et al.*, 2022; Zeng *et al.*, 2022; Zareii *et al.*, 2023; Sun *et al.*, 2025).

**Table 1.** Eligible studies for meta-analysis assessing the association between non-operated endometrioma diameters and serum AMH levels.

Author	Publication site	Country	Design	Number of patients			Age ± SD (years)	Ovarian reserve tests	AMH assay
				Study group	Uni lateral	Bi lateral	Study group		
Hirokawa *et al.* (2011)	*Hum Reprod*	Japan	Prospective observational	38	20	18	33.8 ± 4.7	AMH	IOT
Celik *et al.* (2012)	*Fertil Steril*	Turkey	Prospective observational	65	46	19	28.4 ± 5.7	AMH, AFC, FSH, LH, E2	DSL
Ergun *et al.* (2015)	*J Minim Invasive Gynecol*	Turkey	Prospective observational	26	ND		26.4 ± 6.7	AMH, FSH, E2	Gen II
Giampaolino *et al.* (2015)	*Eur J Obstet Gynecol Reprod Biol*	Italy	Prospective observational	48	48	–	31.7 ± 3.3	AMH	DSL
Sandya and Kumar (2016)	*Int J Infertil Fetal Med*	India	Prospective observational	41	35	6	29.6 ± 3.2	AMH, AFC	‘Immune Chemi luscence’
Mehdizadeh Kashi *et al.* (2017)	*Int J Gynecol Obstet*	Iran	Prospective observational cohort	68	45	23	29.6 ± 5.56	AMH	DSL
Wang *et al.* (2019)	*Gynecol Endocrinol*	Germany	Prospective observational cross-sectional	171	107	64	20–42*	AMH	IOT
Karadağ *et al.* (2020)	*J Obstet Gynaecol*	Turkey	Prospective observational cross-sectional	80	50	30	27.71 ± 4.14	AMH, AFC, FSH, E2	Gen II
Yoon *et al.* (2020)	*Ginekol Pol*	Korea	Retrospective cross-sectional	118	38	80	30.45 ± 5.99	AMH	AMH/MIS EIA
Roman *et al.* (2021)	*Reprod Biomed Online*	France	Prospective observational cohort	220	ND		32.5 ± 5.33	AMH	MIS ELISA, GEN II, A.A.AMH, Elecsys (ELICA)
Akgul *et al.* (2022)	*J Coll Physicians Surg Pak*	Turkey	Retrospective observational controlled	82	82	–	29.32 ± 5.01	AMH	ELICA
Bourdon *et al.* (2022)	*Reprod Biomed Online*	France	Retrospective observational cohort	238	ND		33.9 ± 4.07	AMH	ND
Zeng *et al.* (2022)	*J Ovarian Res*	China	Retrospective cohort	104	ND		33.92 ± 4.08	AMH, AFC, FSH, LH, E2	ND
Zareii *et al.* (2023)	*BMC Women’s Health*	Iran	Retrospective observational cohort	70	32	38	30.7 ± 4.4	AMH	ND
Javedani Masroor *et al.* (2015)	*Middle Eeat Fertil Soc J*	Iran	Prospective observational cross-sectional	57	57	–	29.2 ± 4.03	AMH, AFC, FSH	ND
Sun *et al.* (2025)	*Front Endocrinol*	China	Retrospective observational cohort	65	ND		34.29 ± 10.61	AMH, AFC, FSH	ND

AMH, anti-Müllerian hormone; A.A.AMH, automated access AMH; AFC, antral follicular count; DSL, Diagnostic Systems Laboratories; E2, estradiol; EIA, enzyme immunoassay; ELICA, Electro Chemi Luminescence Immuno Assay; ELISA, enzyme-linked immunoabsorbent assay; GEN II, second generation ELISA; IOT, Immunotech; MIS, Müllerian inhibiting substance; ND, not disclosed.

* Age range (mean was not disclosed).

All eligible studies compared intact (non-operated) endometrioma diameters with serum AMH levels within the same setting. Ten eligible studies were prospective (Hirokawa *et al.*, 2011; Celik *et al.*, 2012; Ergun *et al.*, 2015; Giampaolino *et al.*, 2015; Javedani Masroor *et al.*, 2015; Sandya and Kumar, 2016; Mehdizadeh Kashi *et al.*, 2017; Wang *et al.*, 2019; Karadağ *et al.*, 2020; Roman *et al.*, 2021), and six were retrospective in design (Javedani Masroor *et al.*, 2015; Mehdizadeh Kashi *et al.*, 2017; Yoon *et al.*, 2020; Bourdon *et al.*, 2022; Zeng *et al.*, 2022; Zareii *et al.*, 2023; Sun *et al.*, 2025). Six studies targeted women with unilateral endometriomas (Giampaolino *et al.*, 2015; Javedani Masroor *et al.*, 2015; Mehdizadeh Kashi *et al.*, 2017; Karadağ *et al.*, 2020; Akgul *et al.*, 2022; Zareii *et al.*, 2023), whereas the others included both unilateral and bilateral endometriomas.

### Risk of bias

The risk of bias of all eligible studies was evaluated using the ROBINS-I tool and is summarized in Table 2. All seven domains were assessed, and the overall judgment was determined by the highest level of bias identified across them. As all included studies were observational, the domain of bias due to deviations from intended interventions was considered not applicable. Overall, two studies were rated as having a low risk of bias and 14 as moderate, indicating that the overall body of evidence provides a moderate to good level of certainty.

**Table 2.** ROBINS-I risk of bias assessment in eligible studies: the impact of Endometrioma diameter on serum anti-Müllerian hormone levels.

Study (author year)	Confounding	Selection	Exposure classification	Deviation from intended intervention	Missing data	AMH measurement	Reporting bias	Overall Risk of bias	
Hirokawa *et al.* (2011)	L	L	M	NA	L	L	L	M	No measurement formula for E size No stratification of E size by laterality
Celik *et al.* (2012)	L	L	M	NA	L	L	L	M	No stratification of E size by laterality
Ergun *et al.* (2015)	L	L	M	NA	L	L	L	M	No stratification of E size by laterality No measurement formula for E size
Giampaolino *et al.* (2015)	L	L	L	NA	L	L	L	L	
Sandya and Kumar (2016)	M	M	M	NA	L	M	L	M	Reproductive age not specified in selection criteria, but age range was 25–38. No stratification of E size by laterality No measurement formula for E size, AMH kit ND, Exclusion was not stated for previous hormone treatment
Mehdizadeh Kashi *et al.* (2017)	L	L	M	NA	L	M	L	M	No measurement formula for E size, 15 were excluded due to pregnancy, loss to follow-up, or lack of histological confirmation. No analysis of whether these exclusions introduced bias.
Wang *et al.* (2019)	M	L	M	NA	L	L	L	M	Exclusion was not stated for previous hormone treatment The mean age was ND, only age range.
Karadağ *et al.* (2020)	L	L	L	NA	L	L	L	L	
Yoon *et al.* (2020)	M	M	L	NA	M	L	L	M	Retrospective No stratification of E size by laterality
Roman *et al.* (2021)	M	L	M	NA	L	L	L	M	Exclusion was not stated for previous hormone treatment No stratification of E size by laterality
Akgul *et al.* (2022)	M	M	L	NA	L	M	L	M	Retrospective study Exclusion was not stated for previous hormone treatment
Bourdon *et al.* (2022)	M	M	M	NA	L	M	L	M	Retrospective study Exclusion was not stated for previous hormone treatment No stratification of E size by laterality
Zeng *et al.* (2022)	M	L	L	NA	L	M	L	M	Retrospective No stratification of E size by laterality AMH kit ND
Zareii *et al.* (2023)	M	M	M	NA	L	M	L	M	Retrospective No stratification of E size by laterality Exclusion was not stated for previous hormone treatment
Javedani Masroor *et al.* (2015)	M	L	L	NA	L	M	L	M	Exclusion was not stated for previous hormone treatment
Sun *et al.* (2025)	M	L	M	NA	L	M	L	M	Retrospective No stratification of E size by laterality No measurement formula for E size AMH kit ND

AMH, anti-Müllerian hormone; E, endometrioma; L, low; M, medium; NA, not applicable; ND, not disclosed.

### Endometrioma diameter calculation

In all eligible studies, ultrasonography (US) was used to diagnose endometriomas and measure their sizes (Table 3). In most cases, transvaginal US was employed; however, transabdominal US, transrectal US, computed tomography (CT) scan, and MRI were used in some cases. As outlined in Table 3, in most studies, the ultrasonographic diagnosis of endometrioma was accomplished using standardized criteria (Moore *et al.*, 2002; Kinkel *et al.*, 2006; Van Holsbeke *et al.*, 2010).

**Table 3.** Imaging and measurement approaches for endometrioma diagnosis and diameter evaluation in eligible studies.

Study	Endometrioma diagnosis methods	US endometrioma criteria/performer	Endometrioma size calculation
Hirokawa *et al.* (2011)	TVUS, MRI	Two or more TVUS	ND
Celik *et al.* (2012)	TVUS, CT scan, MRI	ND	Mean of 2 dimensions
Ergun *et al.* (2015)	TVUS	ND	ND
Giampaolino *et al.* (2015)	TVUS	Same investigator	Mean of 3 dimensions
Sandya and Kumar (2016)	TVUS	Same investigator	ND
Mehdizadeh Kashi *et al.* (2017)	TVUS	Standardized criteria/same investigator	ND
Wang *et al.* (2019)	TVUS	ND	Mean of 2 dimensions
Karadağ *et al.* (2020)	TVUS, TRUS	Standardized criteria	Mean of 3 dimensions
Yoon *et al.* (2020)	TVUS, TRUS	ND	Mean of 2 dimensions
Roman *et al.* (2021)	TVUS, TRUS, MRI	Standardized criteria	largest dimension
Akgul *et al.* (2022)	TVUS, TAUS	Standardized criteria	largest dimension
Bourdon *et al.* (2022)	TVUS, MRI	Standardized criteria	largest dimension
Zeng *et al.* (2022)	TVUS, MRI	Standardized criteria	largest dimension
Zareii *et al.* (2023)	US	Skilled gynecologist	ND
Javedani Masroor *et al.* (2015)	TVUS	ND	Mean of 2 dimensions
Sun *et al.* (2025)	TVUS	Standardized criteria	ND

CT scan, computed tomography scan; MRI, magnetic resonance imaging; ND, not disclosed; TAUS, transabdominal ultrasound; TRUS, transrectal ultrasound; TVUS, transvaginal ultrasound; US, ultrasound.

Two main methods were used to measure endometrioma diameter in the included studies. The first method involved averaging two or three perpendicular measurements (Celik *et al.*, 2012; Giampaolino *et al.*, 2015; Javedani Masroor *et al.*, 2015; Wang *et al.*, 2019; Karadağ *et al.*, 2020; Yoon *et al.*, 2020), while the second method recorded the largest single dimension (Roman *et al.*, 2021; Akgul *et al.*, 2022; Bourdon *et al.*, 2022; Zeng *et al.*, 2022). As shown in Table 3, six studies did not disclose their measurement methodology.

### Anti-Müllerian hormone assay methodology

The AMH assays used across the included studies varied, likely reflecting differences in commercial availability and the timing of the study conduct. Details on assay types and their technical performance are summarized in Table 4. Five studies adhered to best-practice protocols for analyte measurement by analyzing all AMH samples in a single batch (frozen samples), thereby minimizing inter-assay variability (Hirokawa *et al.*, 2011; Celik *et al.*, 2012; Ergun *et al.*, 2015; Giampaolino *et al.*, 2015; Wang *et al.*, 2019). In contrast, the other studies appeared to perform AMH measurements on a rolling basis as samples became available (fresh samples). There were no discernible differences in the direction or magnitude of findings between studies employing batch analyses and those using rolling measurements.

**Table 4:** AMH assay kits employed by the eligible studies

Study	Name of AMH assay	Company	Country	Intra-coefficient Of variation	Inter-coefficient Of variation	Lowest Detection Limit ng/ml	Fresh Or Frozen sample
Hirokawa *et al.* (2011)	EIA AMH	IOT	Marseille, France	<12.3%	<14.2%	ND	Frozen*
Celik *et al.* (2012)	ELISA	DSL	ND	4.57%	ND	0.006	Frozen*
Ergun *et al.* (2015)	ELISA GENII	Beckman Coulter	Chaska MN,USA	5.4%	5.6%	ND	Frozen*
Giampaolino *et al.* (2015)	ELISA	DSL	Webster TX, USA	<4.6%	<8%	0.006	Frozen*
Sandya and Kumar (2016)	‘Immune Chemi luscence’	ND	ND	ND	ND	ND	Fresh
Mehdizadeh Kashi *et al.* (2017)	ELISA	DSL	Webster TX, USA	ND	ND	0.006	Fresh
Wang *et al.* (2019)	EIA AMH/MIS	IOT	Chantilly VA, USA	<12.3%	<14.2%	0.006	Frozen*
Karadağ *et al.* (2020)	ELISA GENII	Beckman Coulter	Brea CA, USA	<5.6%	<5.6%	0.0017	Fresh
Yoon *et al.* (2020)	EIA AMH/MIS	IOT	Marseille, France	<12.3%	<14.2%	ND	Fresh
Roman *et al.* (2021)	ELISA 4 different assays	Ansh labs, Beckman Coulter, Roche	USA, Germany	ND	ND	ND	Fresh
Akgul *et al.* (2022)	ELICA	Roche Diagnostics	USA	ND	ND	ND	Fresh
Bourdon *et al.* (2022)	ND	ND	ND	ND	ND	ND	Fresh
Zeng *et al.* (2022)	ND	ND	ND	ND	ND	ND	Fresh
Zarii *et al.* (2023)	ND	ND	ND	ND	ND	ND	Fresh
Javedani Masroor *et al.* (2015)	ND	ND	ND	ND	ND	ND	Fresh
Sun *et al.* (2025)	ND	ND	ND	ND	ND	ND	Fresh

AMH, anti-Müllerian hormone; EIA, enzyme immunoassay; ELICA, Electro Chemi Luminescence Immuno Assay; ELISA, enzyme-linked immunosorbent assay;  DSL, Diagnostic Systems Laboratories; GEN II, second generation ELISA; IOT, Immunotech; MIS, Müllerian inhibiting substance; ND, not disclosed.

* Frozen was selected as it was explicitly noted in the article’s methods.

In five studies, the AMH assay methodology was not explicitly described (Javedani Masroor *et al.*, 2015; Bourdon *et al.*, 2022; Zeng *et al.*, 2022; Zareii *et al.*, 2023; Sun *et al.*, 2025). In one study, multiple assays were used, all employing high-sensitivity ELISA techniques (Roman *et al.*, 2021).

### Primary multivariable cohort-level meta-regression analysis

The primary inferential analysis was a multivariable cohort-level meta-regression incorporating 30 independent cohorts derived from the 16 eligible studies (Supplementary Table S1). This modeling strategy preserved statistical independence across cohorts and enabled adjustment for prespecified covariates.

Endometrioma diameter was not independently associated with serum AMH concentrations. When modeled as a continuous variable, cyst diameter was not a significant predictor of AMH levels (*β* = 0.122, SE = 0.128, *P *= 0.35). Similarly, when categorized into predefined diameter strata, including <4 cm (n = 241), 4–5 cm (n = 218), 5–6 cm (n = 242), and >6 cm (n = 206), no graded or threshold association with AMH depletion was observed (*β* = 0.245, SE = 0.220, *P *= 0.28).

In contrast, maternal age emerged as the dominant determinant of AMH variability, accounting for 58.2% of the between-cohort heterogeneity (*R*^2^ = 58.2%). Endometrioma size contributed negligibly to the explained variance in either modeling strategy.

These findings were consistent across both continuous and categorical specifications of diameter, indicating that the absence of association between cyst size and ovarian reserve is independent of age and robust to alternative model parameterizations.

### The impact of laterality on age and AMH levels

Three studies focused on unilateral endometrioma (Giampaolino *et al.*, 2015; Javedani Masroor *et al.*, 2015; Akgul *et al.*, 2022), three additional provided data on unilateral and bilateral cases separately (Mehdizadeh Kashi *et al.*, 2017; Karadağ *et al.*, 2020; Zareii *et al.*, 2023), while 10 studies presented their data for unilateral and bilateral endometrioma combined (Hirokawa *et al.*, 2011; Celik *et al.*, 2012; Ergun *et al.*, 2015; Sandya and Kumar, 2016; Wang *et al.*, 2019; Karadağ *et al.*, 2020; Roman *et al.*, 2021; Bourdon *et al.*, 2022; Zeng *et al.*, 2022; Sun *et al.*, 2025).

To examine whether endometrioma laterality impacted age and serum AMH levels, we compared six studies with data on unilateral endometrioma to those with data on bilateral cases and those with data on both unilateral and bilateral endometriomas combined.

The two independent sample *t*-test indicated that there was no significant age difference between the two groups (unilateral compared to uni- and bilateral combined), with a mean difference of 1.517 years (95% CI: −4.30 to 2.20, t_13_ = −0.70, *P *= 0.48). In addition, no significant effect of endometrioma laterality on serum AMH levels was detected, with a mean difference of 0.529 ng/ml (95% CI: −1.05 to 2.11, t_14_ = −0.373, *P *= 0.74).

### Secondary and exploratory subgroup analyses

To evaluate the robustness of the primary findings and explore potential non-linear or threshold effects, predefined diameter cut-offs (3, 4, 5, 6, 7, and 8 cm) were examined. As these comparisons repeatedly draw on overlapping cohorts, they were prespecified as supportive and exploratory. Multiplicity was controlled using the Holm–Bonferroni sequentially rejective procedure to maintain a stringent family-wise error rate.

To reduce between-study heterogeneity and assess the consistency of findings across clinically and methodologically relevant strata, subgroup analyses were conducted according to key study characteristics derived from the 16 eligible studies.

Analyses were restricted to prospective designs to evaluate methodological rigor; to unilateral endometriomas to minimize confounding by bilateral ovarian involvement; and to distinct study populations (infertility-only vs unselected endometrioma cohorts) to account for potential baseline differences in ovarian reserve.

Methodological sources of variability were further examined by stratifying according to AMH assay specification, sample processing strategy (single-batch frozen sera vs fresh samples analyzed on a rolling basis), and endometrioma measurement approach (largest single diameter vs averaged orthogonal dimensions). These analyses allowed assessment of whether laboratory methodology or cyst measurement technique contributed materially to heterogeneity in reported associations.

Across all prespecified subgroup domains (prospective designs, unilateral disease, infertility-only cohorts vs unselected endometrioma populations, AMH assay disclosure, frozen vs fresh sample analysis, and endometrioma measurement approach), the direction and magnitude of effects were generally small and inconsistent across thresholds, and none of the subgroup findings materially altered the primary inference that endometrioma diameter is not independently associated with AMH. After Holm–Bonferroni adjustment within each subgroup family, all pooled comparisons remained non-significant, including prospective-only analyses (smallest unadjusted *P *= 0.24; Holm–Bonferroni-adjusted *P *= 0.96), unilateral-only analyses (adjusted *P *= 1.00 for all feasible thresholds), infertility-only analyses (adjusted *P *= 0.75 for all feasible thresholds), AMH assay-disclosed analyses (adjusted *P *= 1.00), and frozen versus fresh/rolling analyses (adjusted *P *= 0.48 and 1.00, respectively, across feasible thresholds). One isolated signal was observed in the measurement-method stratum (largest single-diameter studies at the 3 cm cut-off: WMD −0.587 ng/ml; unadjusted *P *= 0.009; Holm–Bonferroni-adjusted *P *= 0.027), but this was not reproduced across other thresholds, occurred in sparse data, and was not supported by the primary independent-cohort meta-regression; accordingly, it is most consistent with methodological heterogeneity or instability of small subgroup analyses rather than a clinically meaningful diameter threshold (full numeric outputs in Table 5).

**Table 5.** Supportive subgroup and threshold meta-analyses of serum AMH levels by endometrioma diameter (random-effects), with heterogeneity metrics and Holm–Bonferroni-adjusted inference.

Diameter thresholds (cm)	No. of studies	Below threshold (n)	Above threshold (n)	WMD	WMD (95% CI)	Unadjusted *P*-value	**Holm—Adj. *P*-value** *	Hetero-geneity (*I*^2^)	Cochrane’s Q *P*-value	Significance *P*-value
**Prospective-only studies**										
3	4	132	194	−0.196	(−0.935 to 0.543)	0.60	1.00	60%	0.08	NS
5	6	138	146	0.182	(0.653 to 1.017)	0.67	1.00	84%	<0.001	NS
6	3	240	65	−0.198	(−1.408 to 1.012)	0.75	1.00	69%	0.04	NS
7	3	118	115	0.827	(−0.545 to 2.198)	0.24	0.96	75%	0.02	NS
**Unilateral endometrioma**										
3	2	35	71	0.211	(−0.418 to 0.841)	0.51	1.00	0%	0.53	NS
4	2	66	65	0.030	(−1.098 to 1.158)	0.96	1.00	72%	0.06	NS
5	3	76	66	0.118	(−0.457 to 0.692)	0.69	1.00	48%	0.15	NS
**Infertility-only women with endometrioma**										
3	3	135	198	−0.303	(−0.826 to 0.219)	0.25	0.75	0%	0.67	NS
5	2	196	90	−0.270	(−0.767 to 0.227)	0.29	0.75	0%	1.00	NS
6	3	305	75	0.319	(0.287 to 0.925)	0.30	0.75	0%	0.38	NS
**Unselected women with endometrioma**										
3	3	119	212	0.043	(−0.838 to 0.924)	0.92	1.00	77%	0.01	NS
4	4	119	190	0.042	(−0.543 to 0.627)	0.89	1.00	59%	0.06	NS
5	6	139	159	0.251	(−0.617 to 1.118)	0.57	1.00	84%	<0.001	NS
6	4	274	93	−0.034	(−0.762 to 0.694)	0.93	1.00	54%	0.09	NS
7	4	155	140	0.526	(−0.350 to 1.401)	0.24	1.00	64%	0.04	NS
8	3	77	137	0.052	(−0.727 to 0.831)	0.90	1.00	38%	0.20	NS
**AMH assay disclosed**										
3	2	106	163	−0.229	(−1.335 to 0.878)	0.68	1.00	79%	0.03	NS
4	3	98	149	−0.163	(−1.057 to 0.731)	0.72	1.00	72%	0.03	NS
5	5	112	131	−0.130	(−0.930 to 0.671)	0.75	1.00	82%	<0.001	NS
6	3	240	65	−0.198	(−1.408 to 1.012)	0.75	1.00	0%	0.04	NS
7	3	118	115	0.827	(−0.545 to 2.198)	0.24	1.00	75%	0.02	NS
8	2	56	96	−0.223	(−1.469 to 1.024)	0.73	1.00	34%	0.22	NS
**Frozen sera**										
5	3	62	87	−0.408	(−1.358 to 0.543)	0.40	0.48	75%	0.02	NS
7	3	118	115	0.827	(−0.545 to 2.198)	0.24	0.48	75%	0.02	NS
**Fresh sera**										
3	6	254	410	−0.077	(−0.576 to 0.423)	0.76	1.00	51%	0.07	NS
4	6	335	316	0.062	(−0.425 to 0.550)	0.80	1.00	60%	0.03	NS
5	5	273	162	0.318	(−0.282 to 0.917)	0.30	1.00	59%	0.04	NS
6	6	565	146	0.160	(−0.350 to 0.670)	0.54	1.00	42%	0.12	NS
8	2	35	125	−0.073	(−1.057 to 0.912)	0.89	1.00	63%	0.10	NS
**Endometrioma measurement—averaged orthogonal dimensions**										
3	2	35	71	0.211	(−0.418 to 0.841)	0.51	1.00	0%	0.53	NS
5	3	88	74	−0.304	(−1.321 to 0.713)	0.56	1.00	91%	<0.001	NS
**Endometrioma measurement—largest single diameter**										
3	2	201	257	−0.587	(−1.028 to −0.147)	0.009	0.027	0%	0.44	S
4	3	256	168	−0.083	(−0.977 to 0.811)	0.86	1.00	64%	0.06	NS
6	3	466	96	−0.241	(−1.364 to 0.883)	0.67	1.00	62%	0.07	NS

* Adjusted *P*-values were calculated using the Holm–Bonferroni step-down procedure to control for the family-wise error rate (FWER) across the meta-analytic outcomes. This method accounts for the increased risk of Type I errors arising from multiple comparisons within the same study population.

CI, confidence interval; WMD, weighted mean difference; S, significant (*P *< 0.05); NS, non-significant (*P *> 0.05).

## Discussion

### Principal findings

This systematic review and meta-analysis, including 16 studies and 1484 women, demonstrates that the diameter of non-operated endometriomas is not independently associated with ovarian reserve, as assessed by serum AMH levels.

In the primary multivariable cohort-level meta-regression incorporating 30 independent non-overlapping cohorts, endometrioma size was not a significant predictor of AMH concentrations, whether modeled as a continuous variable or categorized into predefined diameter strata (<4 cm, 4–5 cm, 5–6 cm, >6 cm). In contrast, age consistently emerged as the dominant determinant of AMH variability, accounting for the majority of between-cohort heterogeneity.

These findings were robust across all prespecified secondary analyses. No association between endometrioma diameter and AMH was observed in analyses restricted to prospective studies, unilateral disease, defined AMH assay methodologies, single-batch frozen versus fresh sample analysis, infertility-only populations, or according to the method used for cyst size measurement. After adjustment for multiplicity using the Bonferroni–Holm procedure, none of the exploratory threshold comparisons altered the primary inference.

Collectively, the evidence indicates that any reduction in ovarian reserve observed in women with endometrioma appears to be independent of cyst diameter. These findings argue against a clinically meaningful mechanical or ‘mass-effect’ contribution to diminished ovarian reserve. Instead, they support the concept that any ovarian reserve impairment in endometriosis reflects underlying biological mechanisms, including chronic inflammation, oxidative stress, cortical fibrosis, and local cytokine-mediated disruption of the ovarian microenvironment (Kitajima *et al.*, 2011; Sanchez *et al.*, 2014; Wu *et al.*, 2019; Wyatt *et al.*, 2023), rather than cyst size *per se*.

Furthermore, these results are conceptually consistent with a previous systematic review and meta-analysis, which reported similar preoperative AMH levels in women with unilateral versus bilateral endometriomas (Younis *et al.*, 2019). This concordance suggests that if any detrimental impact of endometriomas on ovarian reserve, it is not determined by size or laterality, but rather reflects a broader pathophysiological effect intrinsic to the disease.

### Strengths and limitations

Our systematic review and meta-analysis have several notable strengths. To our knowledge, this is the first comprehensive quantitative synthesis specifically designed to evaluate whether endometrioma diameter influences ovarian reserve, employing strict methodology and serum AMH as the primary outcome. AMH is considered the most sensitive and clinically reliable biomarker of ovarian reserve in the context of endometrioma surgery (Younis *et al.*, 2022).

A key strength of our analysis is its deliberate focus on studies assessing AMH levels in non-operated ovaries, thereby avoiding the confounding effects of surgical intervention on ovarian reserve. Furthermore, we included only studies that evaluated serum AMH concentrations across different endometrioma diameters within the same setting, allowing for internally controlled comparisons.

Our literature search was extensive and systematic, employing three search engines and two major database platforms. This thorough approach enabled the identification of 16 eligible studies comprising a total of 1484 cases. Notably, quality assessment revealed a low to moderate risk of bias in eligible studies, further strengthening the reliability and validity of our findings (Schünemann *et al.*, 2019).

While one prior review reported a negative association between endometrioma size and AMH levels, it was a narrative review and did not quantitatively assess the impact of cyst diameter (Muzii *et al.*, 2023). Previous attempts to address this clinical question have been confined to individual observational studies, many of which have reported inconsistent or conflicting results (Kasapoglu *et al.*, 2018; Marcellin *et al.*, 2019; Somigliana *et al.*, 2020; Roman *et al.*, 2021). In contrast, our analysis integrates data from multiple studies across diverse populations and methodological designs, providing a more robust and generalizable assessment of this potential relationship.

Our systematic review and meta-analysis have several limitations. First, only six of the included studies examined women with unilateral endometriomas, whereas the remaining studies combined unilateral and bilateral cases. Notably, the mean age and serum AMH levels were comparable between these two groups. Furthermore, our subgroup analysis of studies limited to unilateral endometriomas yielded consistent findings, showing that endometrioma size, above or below various diameter thresholds, did not significantly impact AMH levels. These results are further supported by a previous systematic review and meta-analysis, which demonstrated that the laterality, as an indirect measure of endometrioma size burden, does not influence preoperative AMH concentrations (Younis *et al.*, 2019). Therefore, while this heterogeneity in laterality represents a methodological limitation, it does not appear to compromise the validity of our overall conclusion that endometrioma size does not significantly affect ovarian reserve as measured by serum AMH levels.

Second, 6 of the 16 included studies employed retrospective designs, which are inherently more vulnerable to selection bias, incomplete data capture, and unmeasured confounding compared with prospective investigations. The inclusion of retrospective data may therefore have contributed to methodological heterogeneity and residual bias. However, when analyses were restricted to the 10 prospective studies (n = 812), the pooled estimates remained directionally and quantitatively consistent with the overall findings. This concordance suggests that the primary conclusions were not materially driven by retrospective study inclusion and supports the robustness of the overall inference despite design-related limitations.

Third, considerable between-study heterogeneity was observed in several supportive threshold-based subgroup analyses. This variability likely reflects unmeasured methodological and clinical differences across studies, including inter-assay variability in AMH measurement, e.g. automated versus manual platforms (Nelson *et al.*, 2015; Li *et al.*, 2021), differences in endometrioma size assessment methodology, operator-dependent variability in ultrasound measurement, and inconsistency in sample processing and storage. Residual confounding related to differences in baseline ovarian reserve, within-cohort age distribution, bilaterality classification, and ovarian volume may also have contributed, despite adjustment for mean age at the cohort level. Importantly, cyst diameter may not fully capture underlying disease burden, such as cortical fibrosis, inflammatory activity, or concomitant deep infiltrating endometriosis, which could vary across populations. These sources of heterogeneity could not be harmonized at the study level and warrant cautious interpretation of secondary pooled estimates. Standardized prospective studies incorporating uniform AMH assays, harmonized ultrasound protocols, and detailed phenotyping of disease severity are needed to further refine these findings.

Fourth, stratification according to the method of endometrioma size measurement revealed a statistically significant association in the subgroup of studies defining cyst size by the largest single diameter at the 3 cm threshold (WMD −0.587 ng/ml; 95% CI −1.028 to −0.147; unadjusted *P *= 0.009; *I*^2^ = 0%). This finding, however, was derived from only two studies and was not reproduced at alternative thresholds (4 or 6 cm) within the same measurement stratum. Importantly, it was also not supported by the primary multivariable cohort-level meta-regression, which incorporated all independent cohorts and adjusted for age. Accordingly, this isolated signal should be interpreted cautiously and is most consistent with sparse-data instability or methodological heterogeneity rather than evidence of a biologically meaningful diameter threshold.

Fifth, this systematic review was restricted to non-operated endometriomas and therefore does not address whether preoperative cyst diameter influences the magnitude of postoperative AMH decline. Prior systematic reviews and meta-analyses have consistently shown that endometriotic cystectomy, particularly with the stripping technique, is associated with a significant reduction in serum AMH levels, reflecting postoperative impairment of ovarian reserve (Raffi *et al.*, 2012; Somigliana *et al.*, 2012; Younis *et al.*, 2019). A recent critical appraisal of nine systematic reviews concluded that moderate- to high-quality evidence supports a sustained negative impact of cystectomy on ovarian reserve for up to 9–18 months, with a more pronounced decline in bilateral compared with unilateral cases (∼57% vs 39.5%) (Younis and Taylor, 2024). However, whether endometrioma size modifies the extent of postoperative ovarian reserve loss remains uncertain. This unresolved question represents an important knowledge gap and warrants prospective studies specifically designed to evaluate the interaction between preoperative cyst diameter and postoperative AMH decline.

### Comparison with existing literature

In general, ovarian reserve can be assessed in cases with endometrioma using ovarian reserve biomarkers, such as serum AMH levels, as in our systematic review, or by evaluating ovarian response to controlled ovarian stimulation (COS) in the IVF setting.

Over the last decade, multiple studies have investigated ovarian response to COS, yielding inconsistent results (Coccia *et al.*, 2014; Benaglia *et al.*, 2017; Ferrero *et al.*, 2017; González-Foruria *et al.*, 2020; Somigliana *et al.*, 2020; Bourdon *et al.*, 2022). Some reports demonstrated an inverse association between endometrioma diameter and both the number of mature follicles and retrieved oocytes (Coccia *et al.*, 2014; Ferrero *et al.*, 2017; González-Foruria *et al.*, 2020; Somigliana *et al.*, 2020), whereas others did not (Benaglia *et al.*, 2017; Bourdon *et al.*, 2022). Importantly, the threshold for defining endometrioma diameter varied among the studies. Furthermore, the majority of studies were retrospective in design and included relatively modest cohorts of 26–101 women (Benaglia *et al.*, 2017; Ferrero *et al.*, 2017; González-Foruria *et al.*, 2020; Somigliana *et al.*, 2020). Some focused specifically on non-operated ovaries with unilateral endometrioma (Coccia *et al.*, 2014; Ferrero *et al.*, 2017; Somigliana *et al.*, 2020), while others did not. Notably, Bourdon *et al.* (2022) conducted a recent single-center, well-designed study with the largest cohort to date (n = 326), stratifying women into five groups based on the largest endometrioma diameter (<2, 2–4, 4–6, 6–8, and ≥8 cm) (Bourdon *et al.*, 2022). They found no significant difference in the number of oocytes retrieved across these size categories, suggesting that endometrioma size does not affect ovarian reserve and supporting our conclusion.

In parallel, several studies have evaluated ovarian reserve through serum AMH levels, but findings have also been contradictory (Hirokawa *et al.*, 2011; Celik *et al.*, 2012; Ergun *et al.*, 2015; Giampaolino *et al.*, 2015; Javedani Masroor *et al.*, 2015; Sandya and Kumar, 2016; Mehdizadeh Kashi *et al.*, 2017; Abd Elhameid *et al.*, 2018; Marcellin *et al.*, 2019; Wang *et al.*, 2019; Karadağ *et al.*, 2020; Yoon *et al.*, 2020; Roman *et al.*, 2021; Akgul *et al.*, 2022; Bourdon *et al.*, 2022; Grigalashvili, 2022; Zeng *et al.*, 2022; Zareii *et al.*, 2023; Sun *et al.*, 2025). Some studies reported a negative association between increasing endometrioma size and serum AMH levels (Ergun *et al.*, 2015; Sandya and Kumar, 2016; Mehdizadeh Kashi *et al.*, 2017; Karadağ *et al.*, 2020; Sun *et al.*, 2025). Others observed no significant effect (Hirokawa *et al.*, 2011; Javedani Masroor *et al.*, 2015; Wang *et al.*, 2019; Yoon *et al.*, 2020; Akgul *et al.*, 2022; Bourdon *et al.*, 2022; Grigalashvili, 2022; Zeng *et al.*, 2022; Zareii *et al.*, 2023), while some reported paradoxically higher serum AMH levels with increasing cyst diameter (Celik *et al.*, 2012; Giampaolino *et al.*, 2015; Abd Elhameid *et al.*, 2018; Marcellin *et al.*, 2019; Roman *et al.*, 2021). Again, the threshold of endometrioma diameter was inconsistent across all studies, and most were limited by a relatively modest sample size (n = 26–104) (Hirokawa *et al.*, 2011; Celik *et al.*, 2012; Ergun *et al.*, 2015; Giampaolino *et al.*, 2015; Javedani Masroor *et al.*, 2015; Sandya and Kumar, 2016; Mehdizadeh Kashi *et al.*, 2017; Karadağ *et al.*, 2020; Akgul *et al.*, 2022; Grigalashvili, 2022; Zeng *et al.*, 2022; Zareii *et al.*, 2023; Sun *et al.*, 2025).

A previous systematic review and meta-analysis evaluating the effect of endometrioma size on ovarian reserve levels, including seven studies with a total of 603 cases, reported no significant change in serum AMH levels between small and large endometriomas (Liu *et al.*, 2025). However, the review had several key methodological limitations. The largest included study (n = 193) was subsequently retracted from publication (Alborzi *et al.*, 2014). Another study was published exclusively in Chinese without an accessible English translation (Jie-Han *et al.*, 2022), which limited its appraisal and reproducibility. Moreover, the review did not exclude participants with a history of prior ovarian surgery and failed to account for laterality, both important confounders in the assessment of ovarian reserve. Most critically, the definitions of ‘large’ versus ‘small’ endometriomas varied across the included studies, with no consistent threshold applied. This lack of standardization introduced substantial methodological heterogeneity, thereby reducing the reliability of the pooled estimates and weakening the overall level of evidence.

Taken together, available studies assessing the effect of endometrioma diameter on ovarian reserve, through ovarian response to COS and serum AMH levels, produced inconsistent and often contradictory results. Due to methodological heterogeneity and limited study quality, there has been no reliable evidence to date supporting a definitive link between endometrioma size and ovarian reserve.

Our present systematic review, rigorously conducted in accordance with the updated PRISMA 2020 guidelines and the Cochrane Handbook for Systematic Reviews, showing a low to moderate risk of bias employing the ROBINS-I tool, clearly demonstrates that any detrimental effect of endometrioma on ovarian reserve is not related to cyst diameter.

The observation that endometrioma diameter does not significantly correlate with serum AMH levels aligns with emerging histopathological evidence. While mechanical stretching and size-related factors have been traditionally blamed for ovarian reserve depletion, recent studies suggest that the extent of cortical fibrosis in the ovarian tissue neighboring the endometrioma is a more critical determinant of follicular loss. Specifically, research has shown that, along with age, the degree of cortical fibrosis is significantly and negatively correlated with serum AMH levels, whereas lesional fibrosis on the cyst itself is not (Nie *et al.*, 2022). This reinforces the notion that if any detrimental impact of endometriomas on the ovary is driven by intrinsic pathophysiological processes, such as chronic inflammation and localized tissue remodeling, rather than the physical dimensions of the cyst.

Persistent uncertainty regarding the clinical relevance of endometrioma diameter has historically shaped management guidelines, although evolving evidence has prompted a substantial paradigm shift. Earlier recommendations from professional societies, including the American Society for Reproductive Medicine (ASRM) and ESHRE, frequently endorsed a size-based threshold for surgical intervention. A widely cited position suggested that endometriomas larger than 4 cm should be excised to alleviate pain or potentially improve fertility outcomes (Practice Committee of the American Society for Reproductive Medicine, 2012; Dunselman *et al.*, 2014).

More recent guidance from these organizations has moved away from rigid diameter-based criteria, particularly in asymptomatic women and those pursuing fertility preservation or ART. The most recent ESHRE guideline (Becker *et al.*, 2022) emphasizes individualized decision-making, recommending that surgical intervention be guided primarily by pain severity, patient age, ovarian reserve, prior treatment history, and reproductive goals, rather than cyst size alone. Notably, ESHRE no longer supports routine excision of endometriomas before ART in the absence of specific clinical indications. Similarly, contemporary ASRM committee opinions acknowledge that although larger cysts (e.g. >4 cm) may occasionally justify surgery for diagnostic clarification or to facilitate oocyte retrieval, the potential detrimental impact of cystectomy on ovarian reserve must be carefully balanced against anticipated benefits.

In this context, our findings, demonstrating no independent association between endometrioma diameter and serum AMH levels, provide quantitative support for this modern, individualized management paradigm. Taken together, the evidence suggests that clinical decision-making should not rely on cyst size as a solitary determinant, but rather integrate symptomatology, reproductive planning, and baseline ovarian reserve within a patient-centered framework.

### Conclusions and wider implications

This systematic review and multivariable cohort-level meta-analysis provide robust evidence that the diameter of non-operated endometriomas is not independently associated with ovarian reserve, as reflected by serum AMH levels. The consistency of findings across prespecified exploratory subgroup analyses further supports the conclusion that any endometrioma-related impairment in ovarian reserve is unlikely to be mediated by cyst size or a simple ‘mass-effect’. Rather, reduced ovarian reserve in affected women may likely reflects intrinsic pathophysiological mechanisms of endometriosis, including chronic inflammation, oxidative stress, and cortical remodeling.

From a clinical perspective, these findings support an individualized management approach. The presence of a large endometrioma alone should not constitute an indication for surgical intervention in reproductive-age women, particularly when preservation of ovarian reserve is a priority. Instead, decision-making should integrate symptom burden, reproductive goals, age, baseline ovarian reserve markers, and informed patient preference.

These data are relevant across several clinical contexts. In ART, cyst diameter alone should not be considered a determinant of ovarian reserve assessment or pre-ART surgical planning in asymptomatic women. Similarly, in the setting of fertility preservation, the presence of an asymptomatic endometrioma does not appear to imply a size-dependent reduction in ovarian reserve, reinforcing the primacy of age and baseline ovarian reserve parameters in counseling. For patients presenting primarily with pain, the decision to proceed with surgery should be guided by symptom severity and overall clinical context rather than adherence to a fixed diameter threshold.

Future prospective studies are needed to determine whether preoperative endometrioma diameter modifies the magnitude of postoperative AMH decline following cystectomy. Clarifying this relationship will be essential for refining fertility-preserving surgical strategies and optimizing individualized care.

## Supplementary Material

hoag048_Supplementary_Data

## Data Availability

The data underlying this article will be shared on reasonable request to the corresponding author.
