# Supplementary material for: Does endometrioma diameter impact serum anti-Müllerian hormone levels? A systematic review and meta-analysis
Source: Hum Reprod Open. 2026 May 22;2026(3):hoag048. doi: 10.1093/hropen/hoag048 (PMC13283431; doi:10.1093/hropen/hoag048)
Supplement: hoag048_Supplementary_Data [file hoag048_supplementary_data.docx]

Supplementary Table S1: Independent non-overlapping cohorts for the primary multi-variable meta-regression.

| **AMH + SD (ng/ml)** | **Age + SD (yrs.)** | **E size + SD**  **(cm)** | **N** | **Author** |
| --- | --- | --- | --- | --- |
| 4.28 ± 1.69 | 33.8 ± 4.7 | 3.75 ± 0.5 | 4 | Hirokawa et al.,  2011 |
| 3.95 ± 2.53 | 33.8 ± 4.7 | 5.3 ± 0.48 | 10 | Hirokawa et al.,  2011 |
| 5.05 ± 3.2 | 33.8 ± 4.7 | 7.82 ± 0.6 | 11 | Hirokawa et al.,  2011 |
| 2.62 ± 1.76 | 33.8 ± 4.7 | 12.73 ± 1.9 | 11 | Hirokawa et al.,  2011 |
| 1.78 ± 1.71 | 28.4 ± 5.7 | 5.87 ± 2.1 | 65 | Celik et al.,  2012 |
| 6.07 ± 1.16 | 31.7 ± 3.3 | 5.89 ± 2.19 | 48 | Giampaolino et al.,  2015 |
| 3.8 ± 3.06 | 29.6 ± 3.2 | 3.5 ± 1.9 | 41 | Sandya and Kumar,  2016 |
| 3.27 ± 2.83 | 29.6 ± 5.56 | 5.98 ± 2.19 | 70 | Mehdizadeh Kashi et al.,  2017 |
| 2.82 ± 1.16 | 27.71 ± 4.14 | 2.46 ± 0.24 | 9 | Karadağ et al.,  2020 |
| 2.69 ± 0.63 | 27.71 ± 4.14 | 3.33 ± 0.79 | 16 | Karadağ et al.,  2020 |
| 2.51 ± 0.56 | 27.71 ± 4.14 | 4.37 ± 0.15 | 10 | Karadağ et al.,  2020 |
| 2.19 ± 0.56 | 27.71 ± 4.14 | 5.27 ± 0.38 | 7 | Karadağ et al.,  2020. |
| 2.15 ± 0.71 | 27.71 ± 4.14 | 7.3 ± 1.76 | 7 | Karadağ et al.,  2020 |
| 1.93 ± 0.59 | 28.3 ± 4.03 | 4.07 ± 1.49 | 30 | Karadağ et al.,  2020 |
| 3.36 ± 2.63 | 30.45 ± 5.99 | 6.52 ± 3.96 | 116 | Yoon et al., |
| 2.56 ± 2.54 | 29.32 ± 5.01 | 4.1 ± 1.12 | 82 | Akgul et al.,  2022 |
| 2.74 ± 2.06 | 34.56 ± 4.1 | 1.39 ± 0.49 | 79 | Bourdon et al.,  2022 |
| 3.37 ± 2.22 | 33.78 ± 3.73 | 2.56 ± 0.3 | 39 | Bourdon et al.,  2022 |
| 3.02 ± 2.7 | 34.69 ± 4.73 | 3.5 ± 0.19 | 27 | Bourdon et al.,  2022 |
| 3.53 ± 2.46 | 33.21 ± 3.92 | 4.32 ± 0.27 | 26 | Bourdon et al.,  2022 |
| 3.53 ± 3.44 | 33.91 ± 4.02 | 5.42 ± 0.43 | 31 | Bourdon et al.,  2022 |
| 3.09 ± 2.34 | 32.56 ± 3.92 | 6.95 ± 0.33 | 21 | Bourdon et al.,  2022 |
| 3.25 ± 2.15 | 33.57 ± 3.93 | 9.75 ± 2.03 | 15 | Bourdon et al.,  2022 |
| 3.58 ± 3.2 | 30.68 ± 4.4 | 4.34 ± 2.04 | 32 | Zareii et al.,  2023 |
| 2.43 ± 2.14 | 30.68 ± 4.4 | 4.97 ± 2.25 | 38 | Zareii et al.,  2023 |
| 1.99 ± 1.22 | 34.29 ± 10.61 | 1.71 ± 0.71 | 11 | Sun et al.,  2025 |
| 1.43 ± 1.11 | 34.29 ± 10.61 | 3.94 ± 0.53 | 15 | Sun et al.,  2025 |
| 1.43 ± 1.69 | 34.29 ± 10.61 | 5.73 ± 0.57 | 11 | Sun et al.,  2025 |
| 1.4 ± 1.5 | 34.29 ± 10.61 | 7.58 ± 0.33 | 8 | Sun et al.,  2025 |
| 1.67 ± 1.92 | 34.29 ± 10.61 | 9.23 ± 0.49 | 17 | Sun et al.,  2025 |

AMH – anti-Müllerian hormone; E – endometrioma; N – number of patients; SD – standard deviation.
